# Pneumomédiastin spontané: à propos de 18 cas

**DOI:** 10.11604/pamj.2018.31.75.15737

**Published:** 2018-10-02

**Authors:** Chaanoun Khadija, Zaghba Nahid, Benjelloun Hanane, Yassine Nabiha

**Affiliations:** 1 Centre Hospitalier Universitaire Ibn Rochd de Casablanca, Maroc

**Keywords:** Pneumomédiastin, emphysème, spontané, douleur thoracique, pathologie du médiastin, Pneumomediastinum, emphysema, spontaneous, chest pain, mediastinal disease

## Abstract

Le pneumomédiastin spontané se définit par la présence d'air au niveau du médiastin en l'absence de cause traumatique ou iatrogène. Son diagnostic repose sur la radiographie thoracique. Le recours à d'autres examens paracliniques, tels que la tomodensitométrie thoracique ou la fibroscopie bronchique ou digestive, s'impose parfois. L'évolution est le plus souvent favorable. Nous rapportons 18 cas de pneumomédiastin spontané, colligés au service des maladies respiratoires du CHU Ibn Rochd de Casablanca entre 2006 et 2017. Il s'agit de 13 hommes et de cinq femmes dont la moyenne d'âge était de 24 ans. La symptomatologie clinique était dominée par la douleur thoracique rétrosternale. Les circonstances de survenue du pneumomédiastin étaient des quintes de toux dans sept cas, une crise d'asthme dans cinq cas, une consommation de narguilé et des vomissements Itératifs dans deux cas chacun, un accouchement et une exacerbation d'origine bactérienne de BPCO dans un cas chacun. L'évolution était favorable dans tous les cas avec une résorption spontanée du pneumomédiastin. Aucune récidive n'est survenue après un recul moyen de 3 ans.

## Abstract (en)

Spontaneous pneumomediastinum is defined as the presence of air in the mediastinum in the absence of traumatic or iatrogenic cause. Diagnosis is based on chest X-ray. Other paraclinical examinations, such as chest tomodensitometry or bronchial or gastrointestinal endoscopy, are sometimes necessary. Outcome is most often favorable. We report 18 cases of spontaneous pneumomediastinum whose data were collected in the Department of Respiratory Diseases at the Ibn Rochd University Hospital in Casablanca between 2006 and 2017. The study involved 13 men and 5 women, with an average age of 24 years. Clinical symptomatology was dominated by retrosternal chest pain. The circumstances of onset of pneumomediastinum were coughing spell in seven cases, asthma crisis in five cases, hookah consumption and iterative vomiting in two cases, childbirth and exacerbation of chronic obstructive pulmonary disease (COPD) of bacterial origin in one case. Outcome was favorable in all cases, with spontaneous resorption of the pneumomediastinum. No recurrence occurred after a minimum follow-up period of 3 years

## Introduction

Le pneumomédiastin spontané est une entité rare qui survient essentiellement chez l'adolescent et l'adulte jeune dont l'évolution est souvent favorable. Le mécanisme physiopathologique du pneumomédiastin spontané reste encore mal défini. Le but de notre travail est d'évaluer les caractères épidémiologiques, cliniques et évolutifs du penumomédiastin spontané.

## Méthodes

Nous rapportons une étude rétrospective portant sur 18 cas de pneumomédiastin spontané colligés au service des maladies respiratoires du CHU Ibn Rochd de Casablanca, sur une période de 11 ans allant de 2006 à 2017. Le diagnostic de pneumomédiastin a été retenu sur la radiographie thoracique. Aucun patient n'a eu de traumatisme thoracique ni de manoeuvre iatrogène susceptible d'engendrer une lésion de l'arbre trachéo-bronchique avant l'apparition du pneumomédiastin.

## Résultats

**Figure 1:**
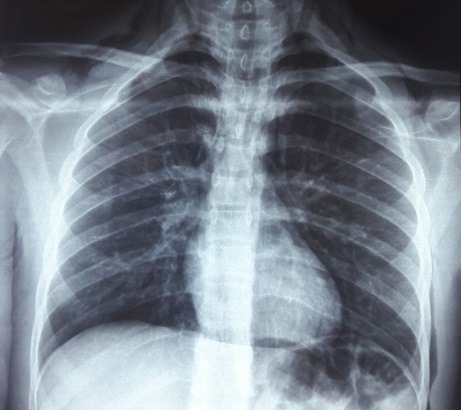
Radiographie thoracique montre un pneumomédiastin, un emphysème des parties molles cervico-thoraciques avec le signe de diaphragme continu

**Figure 2:**
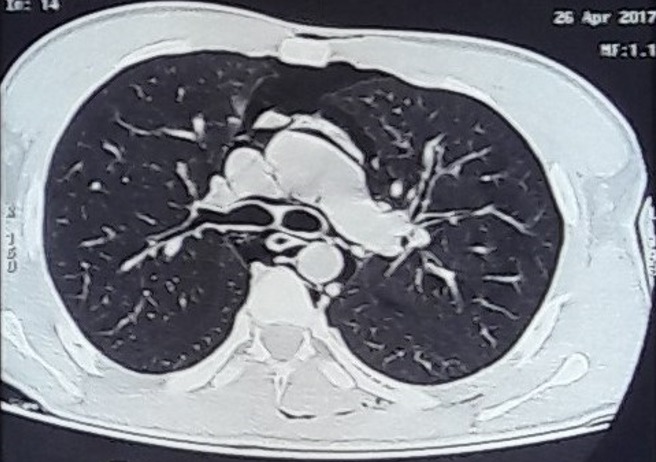
TDM thoracique révélant un pneumomédiastin spontané associé à un emphysème des parties molles cervico thoraciques suite à une crise d’asthme

**Figure 3:**
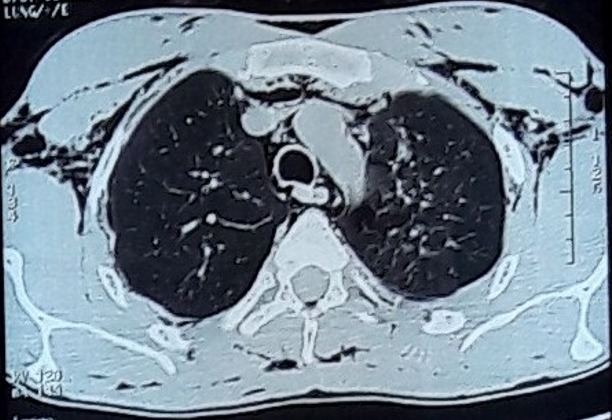
Exacerbation de broncho-pneumopathie chronique obstructive compliquée d’un pneumomédiastin associé un emphysème des parties molles

**Figure 4:**
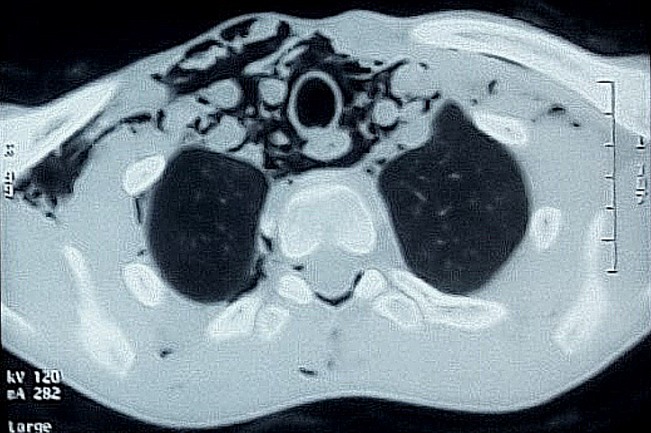
TDM thoracique montant un pneumomédiastin, un pneumopéricarde, un pneumorachis et un emphysème des parties molles chez un patient âgé de 19 ans lors d’un effort de vomissements itératifs

Il s'agissait de 13 hommes et cinq femmes, dont la moyenne d´âge était de 24 ans avec des extrêmes allant de 16 et 57 ans. Dans les antécédents, l'asthme était retrouvé dans quatre cas, la rhinite allergique dans trois cas. Cinq patients étaient tabagiques, dont deux étaient consommateurs de narguilé et un consommateur de cannabis. L'antécédent de tuberculose pulmonaire était retrouvé chez un seul patient. Les circonstances de survenue du pneumomédiastin étaient des quintes de toux dans sept cas, une crise d'asthme dans cinq cas, une consommation de narguilé et des vomissements Itératifs dans deux cas chacun, un accouchement et une exacerbation d'origine bactérienne de BPCO dans un cas chacun. La symptomatologie clinique était dominée par la douleur thoracique survenant chez 14 patients, la dyspnée chez huit patients et la toux sèche chez 4 patients. L'examen clinique révélait un emphysème sous-cutané cervico-thoracique avec crépitations neigeuses chez tous les patients, des râles sibilants chez quatre cas et un syndrome d'épanchement aérique dans trois cas. Le signe de Hammam, n'a été retrouvé que chez trois patients. La radiographie thoracique a objectivé un pneumomédiastin et un emphysème des parties molles cervico-thoraciques dans tous les cas, associés à un pneumothorax dans trois cas (Figure 1). La tomodensitométrie thoracique réalisée chez huit patients a permis de confirmer le diagnostic de pneumomédiastin et de mettre en évidence un pneumorachis cervico-dorsal dans trois cas, un pneumopéricarde dans deux cas et des bulles d'emphysème bilatérales avec un foyer de condensation dans un cas (Figure 2, Figure 3, Figure 4). Afin d'éliminer une lésion de l'arbre trachéo-bronchique ou un obstacle endobronchique, une bronchoscopie souple réalisée chez cinq patients était normale. La prise en charge consistait à un traitement symptomatique, repos et traitement antalgique avec surveillance clinique et radiologique. Un traitement de la crise d'asthme a été entrepris chez les patients asthmatiques et une antibiothérapie, une corticothérapie et un traitement de fond chez patient admis pour exacerbation de la BPCO. La surveillance des patients était pluriquotidienne et reposait, en particulier, sur la fréquence respiratoire, la fréquence cardiaque, la tension artérielle, la saturation en oxygène, la température et la dysphagie. L'évolution était favorable dans tous les cas avec résorption spontanée du pneumomédiastin, du pneumorachis et du pneumopéricarde. La durée moyenne d'hospitalisation était de 8 jours avec des extrêmes allant de 3 à 17 jours. Le recul moyen de surveillance était de 3 ans (extrêmes : 4 mois et 10 ans) sans aucune récidive à distance.

## Discussion

Le pneumomédiastin spontané est une pathologie rare, décrite pour la première fois en 1939 par Hamman [1] dont la présentation clinique initiale peut évoquer une rupture du tractus aéro-digestif. Le mécanisme du pneumomédiastin spontané est encore mal défini et l'hypothèse la plus souvent rapportée dans la littérature est celle d'une hyperpression endo-bronchique à glotte fermée, due à des manœuvres de Valsalva [2, 3]. Cette hyperpression serait responsable d'une rupture alvéolaire et donc l'air va passer dans les espaces interstitiels vers le médiastin en progressant le long des axes broncho-vasculaires jusqu'au hile, puis vers les tissus sous cutanés et les espaces cervicaux profonds et éventuellement vers le péricarde et l'espace épidural à travers les trous de conjugaison [2, 4]. La brèche alvéolaire peut également siéger en périphérie, à travers la plèvre viscérale, créant un pneumothorax associé. L'hyperpression à glotte fermée peut être secondaire à une obstruction bronchique aigue, l'exemple de la crise d'asthme et l'inhalation de corps étranger [5, 6]; ou en cas de ventilation mécanique utilisant de grands volumes et/ou des valeurs élevées de pression en fin d'expiration; ou encore lors d'une toux quinteuse ou d'efforts de vomissement [7, 8]; et enfin lors d'une manœuvre de Valsalva comme la défécation, la manœuvre d'Hemlich ou de l'inhalation volontaire de cocaïne, de marijuana ou d'ecstasy [9, 10]. Un deuxième mécanisme du pneumomédiastin spontané fait appel à des lésions directes des parois alvéolaires [11, 12]. C'est le cas des pneumopathies bactériennes essentiellement à staphylocoques dorés, virales (grippe, rougeole, coqueluche) et parasitaires notamment le cas de la pneumocystose chez les patients immunodéprimés [11, 13]. La miliaire tuberculeuse peut se compliquer aussi d'un pneumomédiastin ainsi que les pneumopathies infiltrantes diffuses au stade de fibrose [12]. Le mécanisme le plus fréquent dans notre série était celui de l'hyperpression à glotte fermé. Le tableau clinique est dominé par les douleurs thoraciques rétro sternales d'installation brutale. La toux est présente chez la moitié des patients et des douleurs cervicales chez un tiers d´entre eux [14]. Dans notre série, la douleur thoracique était le symptôme le plus fréquent, rapporté chez 75% des patients. La dyspnée, la dysphagie et la fièvre sont des signes d'alerte et doivent faire évoquer un pneumomédiastin secondaire à une rupture du tractus aéro-digestive dont l'évolution se fait vers la médiastinite qui constitue une urgence diagnostique et thérapeutique. L'examen clinique a retrouvé un emphysème des parties molles chez tous nos patients, alors que le signe de Hammam n'est retrouvé que chez 19 % des patients, c'est un signe pathognomonique du pneumomédiastin sous forme de bruits bulleux synchrones des battements cardiaques [1]. La radiographie thoracique mis en évidence des clartés fines linéaires intra médiastinales limitées en dehors par la plèvre qui apparaît alors comme un fin liseré opaque. L'air délimite alors le bouton aortique, les reliquats thymiques et l´aorte descendante [15, 16]. Le signe de diaphragme continu et du thymus volant chez l'enfant sont des signes caractéristiques. Parfois, le pneumomédiastin n'est visible que sur le cliché de profil, se traduisant par une clarté rétro-sternale ou des lignes claires bordant la crosse de l'aorte ou délimitant la paroi antérieure de la trachée. La radiographie thoracique permet aussi de mettre en évidence un emphysème des parties molles cervico-thoraciques ou un pneumothorax associés [17]. Cependant, Kaneki trouve que 30 % des pneumomédiastins sont méconnus par la radiographie thoracique seule [18]. Le pneumomédiastin spontané est une pathologie bénigne dont l'évolution spontanée se fait vers la résorption spontanée en 48 à 96 heures par passage direct de l'air dans la circulation sanguine. Le recours aux examens complémentaires est donc rarement nécessaire. La tomodensitométrie thoracique montre une dissection par l'air de l'ensemble des structures anatomiques du médiastin et du cou, elle est indiquée en cas de suspicion de lésion organique associée bronchique ou œsophagienne [15, 18]. Le Transit œsophagien aux hydrosolubles est Indiqué en cas de suspicion de rupture œsophagienne et montre une extravasation du produit de contraste. La prise en charge du pneumomédiastin spontané repose sur un traitement symptomatique. Cependant de rares cas de pneumomédiastins compressifs ont été décrits [19] avec tableau de tamponnade nécessitant un drainage chirurgical. Les récidives sont très rares [16, 20].

## Conclusion

Le pneumomédiastin spontané est une affection bénigne d'évolution souvent favorable dont la prise en charge repose sur un traitement symptomatique. Le seul examen complémentaire nécessaire pour poser le diagnostic positif est la radiographie thoracique. Le recours à d'autres examens complémentaires s'impose parfois pour éliminer un pneuumomédiastin secondaire à une rupture du tractus aéro-digestif.

### Etat des connaissances actuelles sur le sujet

- Le pneumomédiastin spontané est rare;
- C'est une pathologie qui concerne essentiellement le sujet jeune dont l'évolution est souvent favorable.

### Contribution de notre étude à la connaissance

- Le mécanisme du pneumomédiastin reste encore mal défini dans la littérature, nous rapportons dans cet article les différentes hypothèses expliquant la survenue d'un pneumomédiastin en dehors de tout traumatisme ou cause iatrogène;
- Notre étude est une étude rétrospective descriptive portant sur 18 cas de pneumomédiastin spontané compliqués d'un pneumorachis dans trois cas et d'un pneumopéricarde dans deux cas;
- La radiographie thoracique est suffisante pour le diagnostic positif du pneumomédiastin, le recours à d'autres examens complémentaires n'est justifié que lorsqu'on suspecte une rupture du tractus aéro-digestif.

## Conflits d’intérêts

Les auteurs ne déclarent aucun conflit d'intérêts.
